# *HHIPL1*, a Gene at the 14q32 Coronary Artery Disease Locus, Positively Regulates Hedgehog Signaling and Promotes Atherosclerosis

**DOI:** 10.1161/CIRCULATIONAHA.119.041059

**Published:** 2019-06-05

**Authors:** Dimitra Aravani, Gavin E. Morris, Peter D. Jones, Helena K. Tattersall, Elisavet Karamanavi, Michael A. Kaiser, Renata B. Kostogrys, Maryam Ghaderi Najafabadi, Sarah L. Andrews, Mintu Nath, Shu Ye, Emma J. Stringer, Nilesh J. Samani, Tom R. Webb

**Affiliations:** 1 Department of Cardiovascular Sciences, University of Leicester and National Institute for Health Research Leicester Biomedical Research Centre, Glenfield Hospital, United Kingdom (D.A., G.E.M., P.D.J., H.K.T., E.K., M.A.K., M.G.N., S.L.A., M.N., S.Y., E.J.S., N.J.S., T.R.W.).; 2 Department of Human Nutrition, Faculty of Food Technology, University of Agriculture in Kraków, Poland (R.B.K).

**Keywords:** atherosclerosis, coronary artery disease, genome-wide association study, hedgehogs, signaling

## Abstract

**Background:**

Genome-wide association studies have identified chromosome 14q32 as a locus for coronary artery disease. The disease-associated variants fall in a hitherto uncharacterized gene called *HHIPL1* (*hedgehog interacting protein*-*like 1*), which encodes a sequence homolog of an antagonist of hedgehog signaling. The function of HHIPL1 and its role in atherosclerosis are unknown.

**Methods:**

HHIPL1 cellular localization, interaction with sonic hedgehog (SHH), and influence on hedgehog signaling were tested. *HHIPL1* expression was measured in coronary artery disease–relevant human cells, and protein localization was assessed in wild-type and *Apoe*^−/−^ (apolipoprotein E deficient) mice. Human aortic smooth muscle cell phenotypes and hedgehog signaling were investigated after gene knockdown. *Hhipl1*^−/−^ mice were generated and aortic smooth muscle cells collected for phenotypic analysis and assessment of hedgehog signaling activity. *Hhipl1*^−/−^ mice were bred onto both the *Apoe*^−/−^ and *Ldlr*^−/−^ (low-density lipoprotein receptor deficient) knockout strains, and the extent of atherosclerosis was quantified after 12 weeks of high-fat diet. Cellular composition and collagen content of aortic plaques were assessed by immunohistochemistry.

**Results:**

In vitro analyses revealed that HHIPL1 is a secreted protein that interacts with SHH and increases hedgehog signaling activity. *HHIPL1* expression was detected in human smooth muscle cells and in smooth muscle within atherosclerotic plaques of *Apoe*^−/−^ mice. The expression of *Hhipl1* increased with disease progression in aortic roots of *Apoe*^−/−^ mice. Proliferation and migration were reduced in *Hhipl1* knockout mouse and *HHIPL1* knockdown aortic smooth muscle cells, and hedgehog signaling was decreased in *HHIPL1*-deficient cells. *Hhipl1* knockout caused a reduction of >50% in atherosclerosis burden on both *Apoe*^−/−^ and *Ldlr*^−/−^ knockout backgrounds, and lesions were characterized by reduced smooth muscle cell content.

**Conclusions:**

HHIPL1 is a secreted proatherogenic protein that enhances hedgehog signaling and regulates smooth muscle cell proliferation and migration. Inhibition of HHIPL1 protein function might offer a novel therapeutic strategy for coronary artery disease.

## Abstract 2

Supplemental Digital Content is available in the text.

**Clinical Perspective**

**What Is New?**

- This is the first investigation of *HHIPL1* (hedgehog interacting protein-like 1), a candidate gene at the chromosome 14q32 coronary artery disease locus identified through genome-wide association studies.
- We show that HHIPL1 is a secreted protein that interacts with sonic hedgehog and is a positive regulator of hedgehog signaling.
- In murine models, HHIPL1 deficiency attenuates the development of atherosclerosis by reducing smooth muscle cell proliferation and migration.

**What Are the Clinical Implications?**

- Our study supports *HHIPL1* as the causal gene at the 14q32 coronary artery disease locus.
- HHIPL1 is a promising therapeutic target that affects a pathogenic mechanism not addressed by current treatments for coronary artery disease.

Over the past decade, genome-wide association studies (GWAS) have identified a large number of loci that associate with increased risk of coronary artery disease (CAD).^1–4^ Remarkably, only approximately one-third are also associated with conventional cardiovascular risk factors,^2^ and many loci contain genes that have not previously been implicated in cardiovascular pathophysiology.^3^ Investigation of the function of these genes and identifying the pathways through which the genetic variants exert their effects might facilitate the development of novel therapeutic strategies for the treatment of CAD.

The CAD-associated variants at the 14q32 locus fall in *HHIPL1* (*Hedgehog interacting* *protein-like 1*), a gene of unknown function that encodes a paralog of the hedgehog signaling regulator HHIP (hedgehog interacting protein).^5,6^

The mammalian hedgehog proteins (SHH [sonic hedgehog], DHH [desert hedgehog], and IHH [Indian hedgehog]) are secreted molecules that exert a concentration- and time-dependent effect on target cells.^7^ Signal transduction is initiated upon binding of a hedgehog ligand to the canonical receptor PTCH1 and PTCH2 (Patched 1 and 2),^8^ which leads to disinhibition of SMO (Smoothened). SMO triggers a complex signaling cascade that regulates the activation of the GLI (glioma-associated oncogene homolog 1) family zinc finger transcription factors (GLI1, GLI2, and GLI3).^7^ GLI activators induce the transcription of target genes primarily involved in cell proliferation, cell survival, and cell fate specification. Among those genes are several components of the hedgehog pathway itself, including PTCH and GLI.^9,10^ HHIP modulates hedgehog signaling activity by binding and inhibiting the action of hedgehog proteins.^5,6,11,12^

Hedgehog signaling is indispensable for normal embryonic development^7^ and plays critical roles in the maintenance of adult progenitor and stem cell populations and in tissue repair after injury.^13^ In the cardiovascular system, hedgehog signaling is essential for early vascular development,^14–16^ vascular remodeling in the yolk sac,^17,18^ arterial-venous identity,^19,20^ development and maintenance of the coronary vasculature,^20,21^ and vessel maturation.^22^ In adults, hedgehog signaling is involved in the maintenance of adult vasculature and for ischemia-induced neovascularization, including after myocardial infarction.^23–27^ The role of hedgehog signaling in atherosclerosis is less well defined. Expression of hedgehog pathway components has been detected in plaques, and inhibition of hedgehog signaling using an antibody that blocks binding of all 3 hedgehog proteins to PTCH1 increased atherosclerosis in *Apoe*^−/−^ (apolipoprotein E deficient) mice.^28,29^

Here, we report the first experimental investigation of HHIPL1 and present evidence that it is a secreted proatherogenic protein that regulates smooth muscle cell proliferation and migration.

## Methods

Upon reasonable request, the data, analytical methods, and study materials will be made available to other researchers for the purposes of reproducing the results. Extended methods are provided in the online-only Data Supplement.

### Reagents and Cell Lines

Plasmids were prepared by the Protein Expression Laboratory (PROTEX) cloning service at the University of Leicester. Immunoprecipitation was performed using GFP-Trap beads (Chromotek). Anti-FLAG (F3165, Sigma), anti-GFP (MA5-15256, Thermo Fisher Scientific), anti-GLI1 (AF3455, R&D Systems), and anti-β-actin (11355703, Thermo Fisher Scientific) were used for immunoblotting. HEK293 cells were purchased from American Type Culture Collection, and human aortic smooth muscle cells (AoSMCs) were purchased from Invitrogen and Thermo Fisher Scientific; peripheral blood mononuclear cells and macrophages were prepared as described previously.^30^ Coronary artery endothelial cells were purchased from PromoCell. SHH-LIGHT2 cells were the kind gift of Professor P.A. Beachy, Stanford University.

### SHH Reporter Assays

Analysis of hedgehog signaling activity was performed with SHH-LIGHT2 cells, a clonal NIH-3T3 cell line stably expressing a Gli-dependent firefly luciferase and constitutive *Renilla* reporters, as previously described^31^ with minor modifications. SHH-LIGHT2 cells were seeded in 96-well plates at a density of 4000 cells per well in 200 µL of DMEM with 10% FBS. Once confluent, cells were cultured for a further 12 to 24 hours before treatment with conditioned media. Conditioned medium was prepared by transfecting HEK293 cells with SHH-GFP, HHIP-FLAG, HHIPL1-GFP, or GFP control plasmid. Twenty-four hours after transfection, medium was changed to DMEM with 0.5% FBS and collected after a further 24 hours for treatment of SHH-LIGHT2 cells. Firefly luciferase and *Renilla* were measured 24 hours after treatment using the Dual-Glo Luciferase assay system (Promega) and read on a Novostar plate reader (BMG LabTech). The hedgehog signaling activity of mouse AoSMCs was measured by co-culturing wild-type or *Hhipl1*^−/−^ cells with SHH-LIGHT2 cells. Three wild-type and 3 *Hhipl*^−/−^ AoSMCs were used for experiments. Cells were seeded at 5000 SHH-LIGHT2 and 2500 AoSMCS per well in 96-well plates in replicates of 3 to 6. When cells reached confluence, medium was changed to DMEM containing 0.5% FBS. Luciferase activity was measured 24 hours later.

### Cellular Assays

Proliferation was determined by incubating with PrestoBlue (Thermo Fisher Scientific) and measuring fluorescence emission or by counting cells. Cell migration was measured using a wound-healing assay. Apoptosis was measured by staining cells with FITC Annexin V (Biolegend) and measured with a Beckman Coulter Gallios flow cytometer.

### Generation of Mouse Models

All work involving animals was approved by the local animal ethics committee and performed according to ARRIVE (Animal Research: Reporting of In Vivo Experiments) guidelines and under United Kingdom Home Office Project Licence (60/4332). All mice were housed in a specific pathogen-free facility in an individually ventilated caging system and were group housed wherever possible, and the health status was checked routinely. Other than weight gain associated with high-fat diet, no mice demonstrated any adverse effects. A genetically altered mouse strain was generated from embryonic stem cells (*Hhipl1*^*tm1a(KOMP)Wtsi*^), purchased from the Knockout Mouse Project (KOMP), by the Gene Editing and Archiving Service (GenEAS) in the University of Leicester Division of Biological Services. All work reported here was conducted on mice carrying the knockout first allele (*Hhipl1*^*tm1a(KOMP)Wtsi*^), which is subsequently referred to as *Hhipl1*^−/−^.

### Analysis of Atherosclerosis

The *Hhipl1*^−/−^ strain was backcrossed onto a C57BL6/J background for 6 generations before being intercrossed with *Apoe*^−/−^ and *Ldlr*^−/−^ (low-density lipoprotein receptor deficient) mice to generate *Hhipl1*^−/−^*;Apoe*^−/−^ and *Hhipl1*^−/−^*;Ldlr*^−/−^ mice and control littermates. Because of genotype requirements, mice could not be randomized into groups. Experiments were powered for *en face* analysis of atherosclerosis as the primary objective. The intercrossed mice were fed a high-fat Western diet (TestDiet 5TJN: fat 40%, carbohydrate 44%, protein 16%, cholesterol 0.15%) for 12 weeks from 6 weeks of age. The aortic roots and thoracic aortas were collected and processed. For *en face* analysis, after overnight fixation in 4% PFA, the thoracic aorta was opened longitudinally and stained with 60% Oil Red O (Sigma-Aldrich) and imaged with a DM2500 Leica microscope. The lipid-stained area was quantified with LAS V 4.0 software by a researcher blinded to genotype and is presented as a percentage of total aortic area. For aortic roots, lesion area was measured on Oil Red O–stained sections from frozen embedded hearts. Ninety 10-μm sections were collected to obtain 900 μm of aortic length from the appearance of the aortic sinus (identified by the appearance of aortic cusps), which was deemed point zero. Quantification of lesion area was performed across 9 sections (100 µm between sections). For *en face* analysis, the data are presented as average lesion area normalized to total aorta area. For aortic root analysis, the data are presented as area.

### Atherosclerotic Lesion Compositional Analysis

Smooth muscle cells were stained with anti-α-smooth muscle actin (SMA; ab5694, Abcam), which detects smooth muscle α-actin 2 (ACTA2), and macrophages were stained using macrophages/monocytes antibody (MOMA-2; MCA519G, Bio-Rad). Slides were fixed in cold acetone at −20°C and air dried, and endogenous peroxidase activity was blocked in 0.3% H_2_O_2_/methanol. Nonspecific binding was reduced by incubation in 2.5% goat serum (Vector Laboratories) before primary antibody incubation at 4°C in a humidified chamber overnight. Slides were incubated with ImmPRESS HRP anti-rabbit (Vector Laboratories, MP-7451) or anti-rat IgG peroxidase (Vector Laboratories, MP-7444), and staining was visualized using DAB and counterstained in hematoxylin (Gill No. 2, Sigma-Aldrich). Images were acquired with a DM2500 Leica microscope. Positively stained areas of each aorta were measured with ImageJ by a researcher blinded to genotype. The data are presented as the average of positively stained lesion areas (9 serial sections) normalized by total lesion area. For collagen and lipid core quantification, sections were stained with Masson’s trichrome (Sigma-Aldrich) according to the manufacturer’s instructions.

### Hhipl1 Staining

Serial frozen aortic root sections from *Apoe*^−/−^ and wild-type mice were stained by immunohistochemistry with anti-HHIPL1 (HPA052767; Atlas Antibodies), MOMA-2, and SMA (ab32575; Abcam) antibodies using the same methodology as before. Immunofluorescence was performed on paraffin-embedded aortic root sections from *Apoe*^−/−^ mice using anti-HHIPL1 antibody and Cy3-conjugated mouse monoclonal anti–α-SMA (1:200 C6198, Sigma-Aldrich) antibodies. Sections were treated with antigen unmasking solution (H-3301, Vector Laboratories). Alexa Fluor 488–labeled goat anti-mouse secondary antibody (Thermo Fisher) was used to detect anti-HHIPL1 antibody localization. Images were acquired with a DM2500 Leica fluorescent microscope. Dual staining was quantified across 3 sections using ImageJ.

### Statistical Analysis

Results are presented as mean±SD unless stated otherwise. We conducted 2-sample unpaired Student *t* tests for variables that were normally distributed and compared between 2 groups. If the variable was not normally distributed, we conducted a Mann–Whitney test. For the SHH reporter, *Hhipl1* expression, and human AoSMC migration data, we used the ANOVA approach to compare >2 groups. For mouse and human AoSMC proliferation data, the ANOVA model also incorporated time and the 2-way interaction effect of group and time. We modeled the body weight of each mouse (measured separately at weekly intervals throughout the study) using a linear mixed model, incorporating genotype and time as fixed effects and mouse as a random effect. We assessed all models for underlying assumptions using appropriate plots and statistics. When multiple comparisons of grouping variables were conducted, we adjusted estimated probabilities by the Tukey method to account for multiple comparisons. All statistical tests were 2-sided with a type 1 error rate (*P* value) of 0.05 to determine statistical significance. All statistical analyses were performed with GraphPad Prism 7.00 (GraphPad Software Inc.) and R software version 3.5 (R Core Team, 2018).

## Results

### HHIPL1 Is a Secreted Positive Regulator of SHH

**Figure 1.**
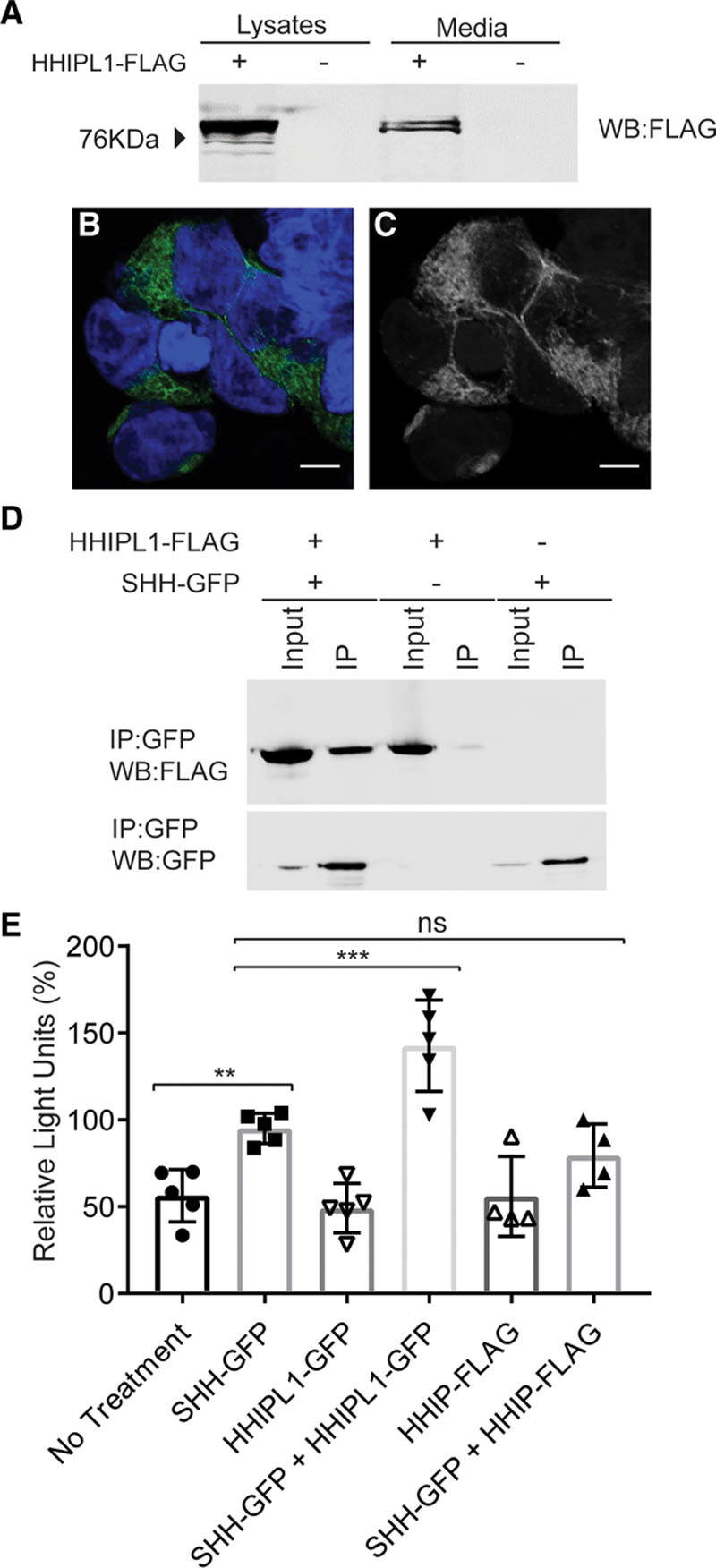
**HHIPL1 is a secreted interactor of SHH**. **A**, Representative Western blot of HEK293 cell lysates and conditioned media after transfection of HHIPL1-FLAG plasmid. **B** and **C**, Representative confocal images of HEK293 cells expressing HHIPL1-GFP (green) costained with DAPI (blue). Bars, 10 μm. **D**, Western blots, immunoblotted with anti-GFP and anti-FLAG antibodies, after transfection of HEK293 cells with HHIPL1-FLAG, SHH-GFP, and immunoprecipitation with anti-GFP beads. **E**, Gli-luciferase activity in SHH-LIGHT2 cells incubated with conditioned media from HEK293 cells transfected with SHH-GFP, HHIPL1-GFP, or HHIP-FLAG or a mixture of SHH-GFP with HHIPL1-GFP or HHIPL-FLAG; n=4–5. GFP indicates green fluorescent protein; HHIPL1, hedgehog interacting protein-like 1; IP, immunoprecipitation; ns, nonsignificant; and SHH, sonic hedgehog. Error bars represent mean±SD. **P≤0.01, ***P≤0.001.

*HHIPL1* encodes a predicted secreted protein consisting of an N-terminal signal peptide followed by a span of 550 amino acids that shares ≈50% identity to the hedgehog interacting region of HHIP and a C-terminal scavenger receptor cysteine-rich domain. We assessed the localization of the HHIPL1 protein by expressing C-terminally FLAG GFP-tagged HHIPL1 expression constructs (HHIPL1-FLAG and HHIPL1-GFP) in HEK293 cells. We detected HHIPL1-FLAG by Western blotting in both HEK293 cell lysates and in conditioned media precipitated with trichloroacetic acid (Figure 1A). No HHIPL1-GFP was seen at the cell surface, which suggests that HHIPL1 is not associated with the cell membrane (Figure 1B and 1C).

Next, we investigated whether HHIPL1 interacts with SHH, the best characterized ligand of the hedgehog pathway. HEK293 cells were cotransfected by electroporation with HHIPL1-FLAG and SHH-GFP plasmids or empty vector controls followed by immunoprecipitation with GFP-Trap beads. Both HHIPL1-FLAG and SHH-GFP fusion proteins were detected in the immunoprecipitated samples, which indicates that HHIPL1 and SHH interact (Figure 1D).

To assess whether HHIPL1 modulates SHH signaling, we performed reporter assays using SHH-LIGHT2 cells.^30^ HHIPL1-GFP in conditioned medium significantly increased Gli-luciferase activity compared with SHH-GFP alone (*P*<0.001; Figure 1E), which suggests HHIPL1 acts as a positive regulator of SHH. HHIP-FLAG caused a nonsignificant reduction compared with SHH-GFP treatment (*P*=0.44).

### *HHIPL1* Is Expressed by Smooth Muscle Cells and Controls Cell Proliferation and Migration

**Figure 2.**
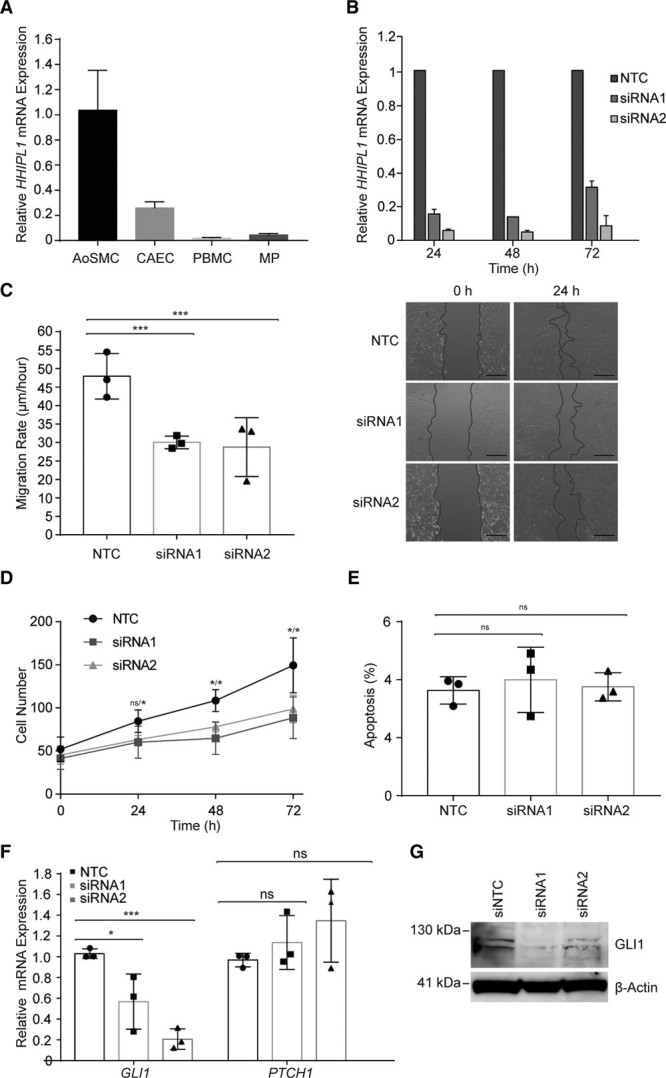
**HHIPL1 regulates human AoSMC migration and proliferation**. **A**,*HHIPL1* mRNA expression in human aortic smooth muscle cells (AoSMC), coronary artery endothelial cells (CAEC), peripheral blood mononuclear cells (PBMC), and macrophages (MP) relative to *36B4*. **B**,*HHIPL1* mRNA expression relative to *RPLP0* in AoSMCs 24 to 72 hours after transfection. **C**,Migration rate of rate of human AoSMCs after siRNA transfection (**left**; n=3). Representative images of wound-healing assay (**right**). **D**, Number of AoSMCs over 72 hours after siRNA knockdown. **E**,Proportion of apoptotic cells 48 hours after knockdown. **F**, *GLI1* and *PTCH1* expression relative to *RPLP0* 48 hours after siRNA knockdown. **G**, Representative Western blot of GLI1 after siRNA knockdown. β-actin was used as a loading control. AoSMC indicates aortic smooth muscle cell; HHIPL1, hedgehog interacting protein-like 1; NTC, nontargeting control siRNA; and siRNA, small interfering RNA.

We measured *HHIPL1* in primary human AoSMCs, coronary artery endothelial cells, peripheral blood mononuclear cells, and macrophages and found the highest expression in AoSMCs (Figure 2A). In addition, the Human Protein Atlas identified high levels of HHIPL1 protein in human smooth muscle cells and breast myoepithelial cells, as well as lower levels in cardiac and skeletal muscle myocytes.^32^

We used small interfering RNA (siRNA) knockdown to investigate the cellular consequences of *HHIPL1* deficiency in human AoSMCs. We used 2 siRNAs to reduce *HHIPL1* expression and achieved knockdown efficiencies between 70% and 95% compared with a nontargeting control siRNA (Figure 2B). Knockdown was retained over a period of 72 hours. We performed a scratch assay to assess the effect of *HHIPL1* deficiency on human AoSMC migration and found that the migration rate of *HHIPL1* knockdown AoSMCs was lower than nontargeting control siRNA transfected cells (siRNA1 *P*<0.001, siRNA2 *P*<0.001; Figure 2C). We assessed the proliferation of *HHIPL1* knockdown cells using a fluorescent cell viability assay and observed a reduction in cell number in *HHIPL1* knockdown cells compared with controls (siRNA1 *P*=0.02, siRNA2 *P*=0.03 at 72 hours; Figure 2D). We detected no difference in apoptosis between control and *HHIPL1* knockdown cells (Figure 2E).

### GLI1 Expression Is Reduced in *HHIPL1*-Deficient AoSMCs

Hedgehog signaling activates gene expression of pathway members including *GLI1* and *PTCH1*. We measured the expression of both genes after *HHIPL1* knockdown in human AoSMCs and detected a significant reduction in *GLI1* expression (siRNA1 *P*=0.041, siRNA2 *P*=0.0002; Figure 2F). We confirmed the reduction in GLI1 protein expression by Western blotting (Figure 2G). We did not detect a difference in *PTCH1* expression. We also assessed *PTCH2* expression, but this was below the threshold for quantification.

### *Hhipl1* Is Present in Smooth Muscle Cells In Vivo, and ItsExpression Increases in Atherosclerosis

**Figure 3.**
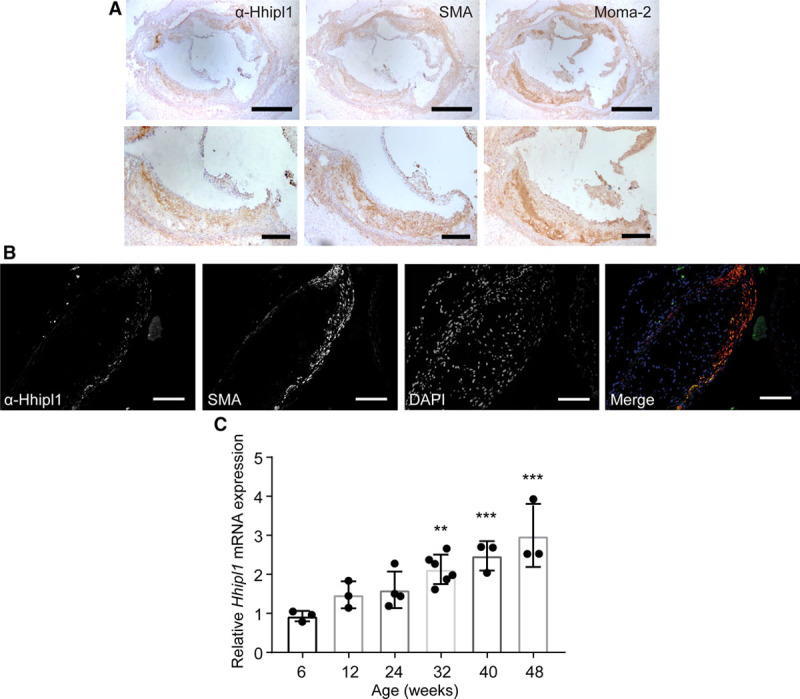
***Hhipl1* expression in atherosclerotic plaques**. **A**, Representative immunohistochemical staining with anti–α smooth muscle actin antibody (SMA), anti-Hhipl1, and MOMA-2 in aortic root lesions from 18-week-old *Apoe*^−/−^ mice fed Western diet for 12 weeks. Bars, 500 μm (**top**) and 200 μm (**bottom**). **B**, Immunofluorescent staining of aortic root lesion with DAPI, SMA, and anti-Hhipl1. Bars, 100 μm. **C**, *Hhipl1* mRNA expression relative to *Rpl4* in the aortic arch of 6- to 48-week-old *Apoe*^−/−^ mice. n=3–6 mice per time point. Error bars represent mean±SD. HHIPL1 indicates hedgehog interacting protein-like 1. *Post hoc comparisons with 6-week time point. **P≤0.01, ***P≤0.001.

We investigated the expression of *Hhipl1* in a hyperlipidemic mouse atherosclerosis model. First, we performed immunohistochemical analysis to detect Hhipl1 protein in aortic root sections from 18-week-old *Apoe*^−/−^ mice fed a high-fat Western diet. Hhipl1 expression most closely matched cells stained with the smooth muscle marker ACTA2, but not those stained with MOMA-2, which recognizes a mouse macrophage antigen (Figure 3A and Figure IA in the online-only Data Supplement). We also detected Hhipl1 expression in aortic arches from wild-type mice (Figure IB in the online-only Data Supplement).

We performed immunofluorescence staining of plaques to confirm expression of Hhipl1 in smooth muscle cells (Figure 3B) and detected Hhipl1 expression in 93.7±2.6% of ACTA2-expressing cells (n=3 cells).

Next, we assessed *Hhipl1* levels during atherosclerosis progression by measuring its expression in RNA collected from the aortic arch of *Apoe*^−/−^ mice between 6 and 48 weeks of age. *Hhipl1* expression increased by approximately 3-fold throughout the study (Figure 3C). Post hoc comparisons to the 6-week time point showed a significant increase in expression at 32 (*P*=0.002), 40 (*P*<0.001), and 48 weeks (*P*<0.001). *Hhipl1* expression did not change in the aortic arch of wild-type mice across the same time frame (Figure IC in the online-only Data Supplement), which indicates that the increase in expression was the result of disease rather than age.

### Generation and Characterization of *Hhipl1* Knockout Mice

We obtained mouse embryonic stem cells carrying the Hhipl1 tm1a knockout first allele, which consists of a reporter-tagged insertion into intron 1 of the *Hhipl1* gene (Figure IIA in the online-only Data Supplement) from the KOMP Repository and generated *Hhipl1*^−/−^ mice. The gene trap is predicted to cause a truncated Hhipl1 protein of just 91 amino acids. We confirmed absence of *Hhipl1* expression in *Hhipl1*^−/−^ mice at the mRNA level by reverse-transcription polymerase chain reaction (Figure IIB in the online-only Data Supplement). *Hhipl1*^−/−^ mice were monitored for signs of subviability and dysmorphology at all stages, but nothing of note was observed. Mice were born in the expected mendelian frequency, and there were no losses perinatally or postnatally. Animals were weighed weekly and showed no difference in weight compared with wild-type littermates.

### *Hhipl1* Knockout AoSMCs Show Reduced Proliferation and Migration

**Figure 4.**
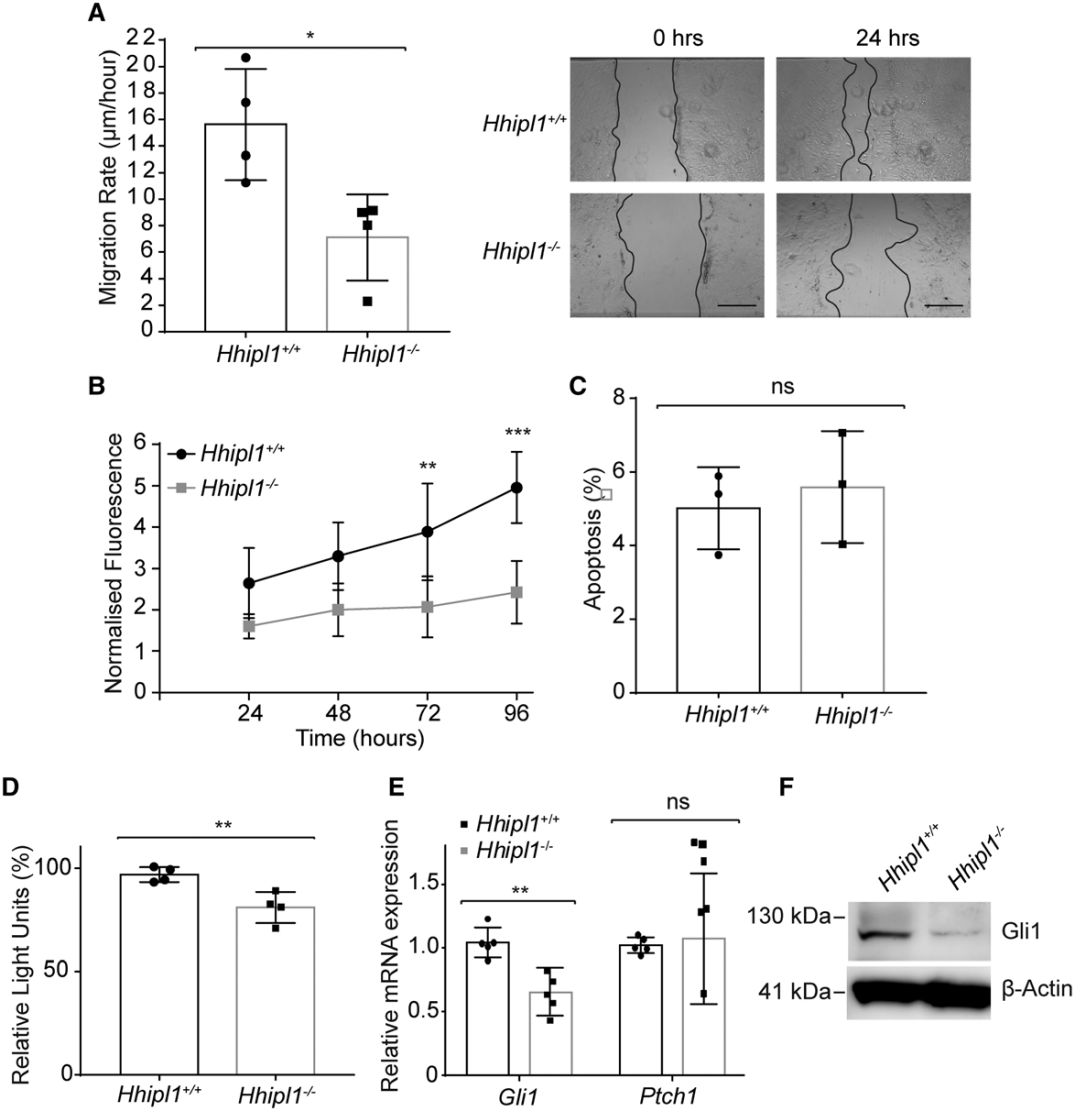
**HHIPL1 regulates mouse AoSMC migration and proliferation**. **A**,Migration rate of *Hhipl1*^−/−^ and wild-type AoSMCs in a scratch wound assay over a period of 24 hours (n=4). Representative images are shown (**right**). **B**, Proliferation of *Hhipl1*^−/−^ and wild-type AoSMCs over a period of 96 hours (n=4). *Significant post hoc comparisons at 72 and 96 hours. **C**, Proportion of apoptotic wild-type and *Hhipl1*^−/−^ AoSMCs. **D**, Gli-luciferase activity in SHH-LIGHT2 cells co-cultured with either wild-type or *Hhipl1*^−/−^ AoSMCs; n=4. **E**, *Gli1* and *Ptch1* mRNA expression relative to *Rplp0* in wild-type and *Hhipl1*^−/−^ AoSMCs; n=5. Error bars represent mean±SD. *P≤0.05, **P≤0.01, ***P≤0.001. **F**, Western blot showing Gli1 expression in wild-type and knockout cells. β-actin was used as a loading control. AoSMC indicates aortic smooth muscle cell; HHIPL1, hedgehog interacting protein-like 1; and SHH, sonic hedgehog.

We collected AoSMCs from knockout mice and wild-type littermates to test the effect of *Hhipl1* deficiency on cell phenotype. Migration (*P*=0.02; Figure 4A) and proliferation (Figure 4B; *P*<0.001 at 96 hours of culture) of *Hhipl1*^−/−^ mouse AoSMCs was decreased compared with wild-type cells. We saw no difference in apoptosis between groups (Figure 4C).

### *Hhipl1* Knockout AoSMCs Have Reduced Hedgehog Signaling Activity

To investigate hedgehog signaling activity, we cultured wild-type and *Hhipl1*^−/−^ mouse AoSMCs together with SHH-LIGHT2 cells and performed luciferase reporter assays. Gli-luciferase activity was significantly reduced in knockout cells (*P*=0.008; Figure 4D). Similar to our experiments in human cells, we detected a reduction in expression of the hedgehog target gene *Gli1* at both the mRNA (*P*=0.02) and protein (Figure 4E and 4F) level. We did not detect a change in *Ptch1* expression in *Hhipl1*^−/−^ AoSMCs.

### *Hhipl1* Knockout Decreases Atherosclerosis in 2 Mouse Models

**Figure 5.**
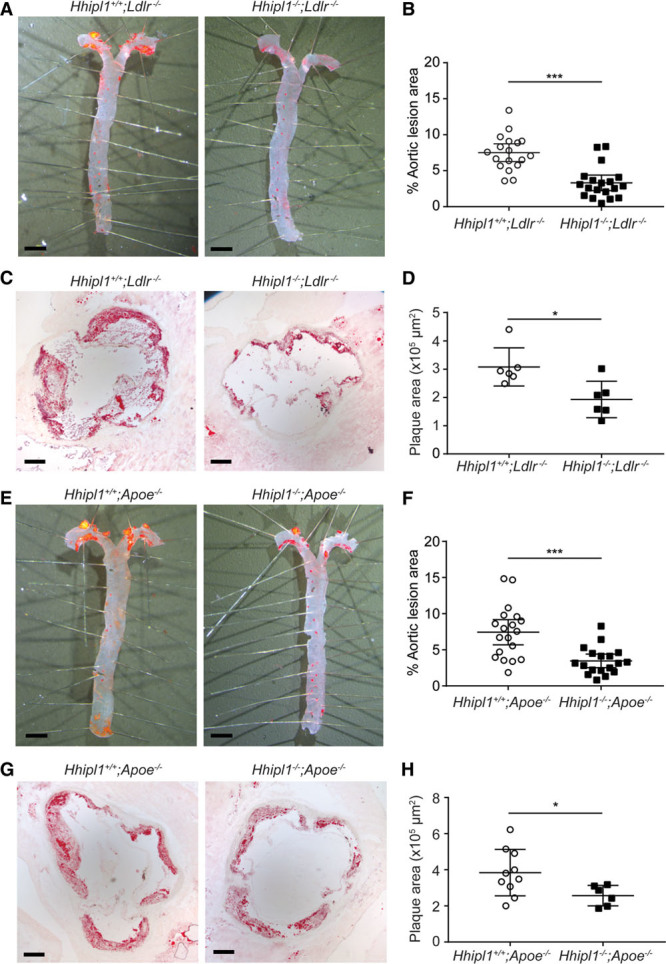
***Hhipl1* deficiency reduces atherosclerosis in *Hhipl1*^−/−^;*Apoe*^−/−^ and *Hhipl1*^−/−^;*Ldlr*^−/−^ mice**. **A**, Representative Oil Red O–stained (ORO) aortas from *Hhipl1*^+/+^;*Ldlr*^−/−^ and *Hhipl1*^−/−^;*Ldlr*^−/−^ mice. **B**, Quantification of atherosclerosis in aortas of mice of each genotype as a percentage of total aorta area (n=18 vs n=19). **C**, Representative microphotographs of ORO-stained aortic root sections from *Hhipl1*^+/+^;*Ldlr*^−/−^ and *Hhipl1*^−/−^;*Ldlr*^−/−^ mice. **D**, Aortic root lesion area (9 sections per mouse, n=6 per group). **E**, ORO-stained aortas from *Hhipl1*^+/+^;*Apoe*^−/−^ and *Hhipl1*^−/−^;*Apoe*^−/−^ mice. **F**, Quantification of atherosclerosis in the aortas of mice of each genotype (n=19 vs n=18). **G**, ORO-stained aortic roots from *Hhipl1*^+/+^;*Apoe*^−/−^ and *Hhipl1*^−/−^;*Apoe*^−/−^ mice. **H**, Aortic root lesion area (9 sections per mouse, n=10 vs n=6). Bars, 2 mm (**A** and **E**); bars, 200 μm (**C** and **G**). Error bars represent mean±CI. HHIPL1 indicates hedgehog interacting protein-like 1. *P≤0.05, ***P≤0.001.

We investigated the effect of *Hhipl1* knockout on atherosclerosis in both *Apoe*^−/−^ and *Ldlr*^−/−^ mice. Male double knockouts (*Hhipl1*^−/−^;*Apoe*^−/−^ and *Hhipl1*^−/−^;*Ldlr*^−/−^) were fed a Western diet for 12 weeks and compared with littermates that were wild type for *Hhipl1* (*Hhipl1*^+/+^;*Apoe*^−/−^ and *Hhipl1*^+/+^;*Ldlr*^−/−^). Atherosclerosis was quantified in the aorta by *en face* analysis (n=18–19 per group) and in sections of the aortic root (n=6–10 per group). *Hhipl1*^−/−^;*Ldlr*^−/−^ mice exhibited a reduction of 56% in lesion area (7.5%; 95% CI, 6.3%–8.7%) compared with *Hhipl1*^+/+^;*Ldlr*^−/−^ littermate controls (3.3%; 95% CI, 2.3%–4.4%) as measured by *en face* analysis (*P*=5×10^−^^6^; Figure 5A and 5B). In aortic roots, there was a 37% reduction in mean lesion area between *Hhipl1*^+/+^;*Ldlr*^−/−^ (3.08×10^5^ µm^2^; 95% CI, 2.37×10^5^ to 3.79×10^5^ µm^2^) and *Hhipl1*^−/−^;*Ldlr*^−/−^ (1.93×10^5^ µm^2^; 95% CI, 1.25×10^5^ to 2.61×10^5^ µm^2^) mice (*P*=0.013; Figure 5C and 5D). Similarly, *Hhipl1*^−/−^;*Apoe*^−/−^ mice displayed a 53% reduction in lesion area (7.5%; 95% CI, 5.7%–9.2%) compared with *Hhipl1*^+/+^;*Apoe*^−/−^ controls (3.5%; 95% CI, 2.5%–4.4%) by *en face* analysis (*P*=0.0002; Figure 5E and 5F). Aortic roots from *Hhipl1*^−/−^;*Apoe*^−/−^ mice showed a decrease of 33% in mean lesion area (3.84×10^5^ µm^2^; 95% CI, 2.92×10^5^ to 4.76×10^5^ µm^2^) versus (2.57×10^5^ µm^2^; 95% CI, 1.97×10^5^ to 3.16×10^5^ µm^2^) compared with controls (*P*=0.039; Figure 5G and 5H).

There was no difference in body weight, plasma lipid levels, or blood pressure between experimental and control groups on either background (Figure IIIA through IIIF in the online-only Data Supplement).

### *Hhipl1* Knockout Reduces Smooth Muscle Cell Content in Mouse Atherosclerotic Plaques

**Figure 6.**
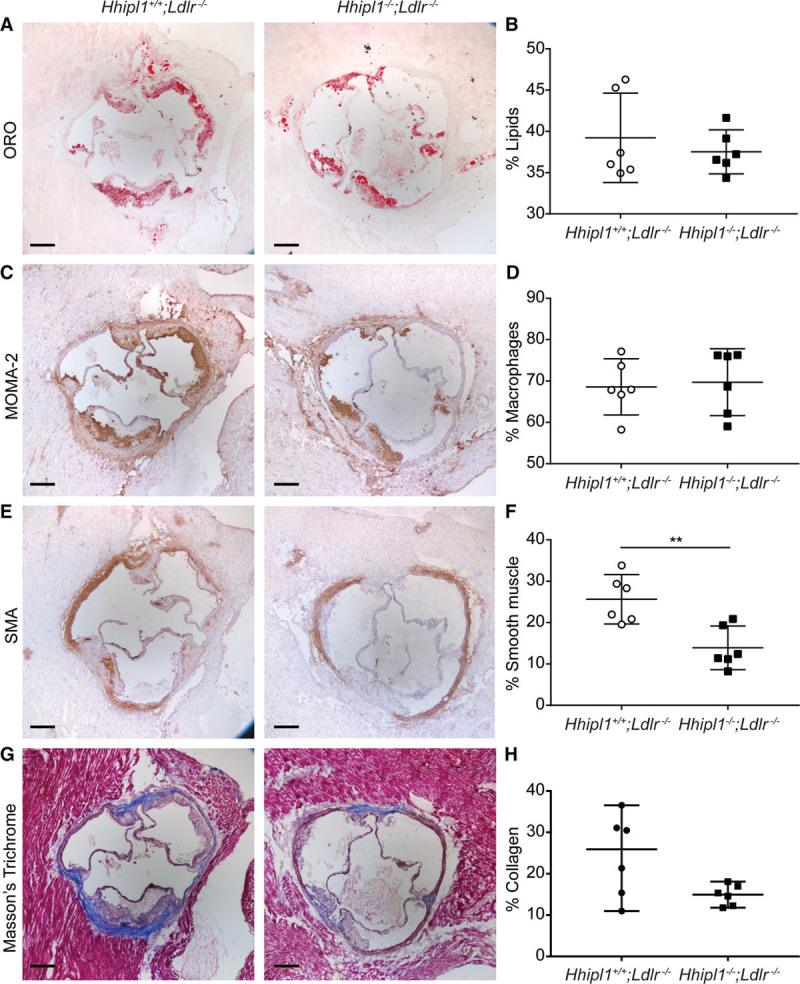
***Hhipl1* deficiency reduces smooth muscle cell content in *Hhipl1***^***−/−***^;***Ldlr***^***−/−***^**atherosclerotic lesions.** Representative photomicrographs of atherosclerotic lesion components in *Hhipl1*^+/+^;*Ldlr*^−/−^ and *Hhipl1*^−/−^;*Ldlr*^−/−^ mice. **A**, Oil red O (ORO) staining for lipids; **C**, MOMA-2 staining for macrophages; **E**, anti–alpha smooth muscle actin (SMA) staining for smooth muscle cells; and (**G**) Masson trichrome for collagen. Bars, 200 μm. The percentage content (average of 9 sections per animal) of (**B**) lipids, (**D**) macrophages, (**F**) smooth muscle cells, and (**H**) collagen (n=6 per group). Error bars represent mean±CI. HHIPL1 indicates hedgehog interacting protein-like 1. **P≤0.01.

**Figure 7.**
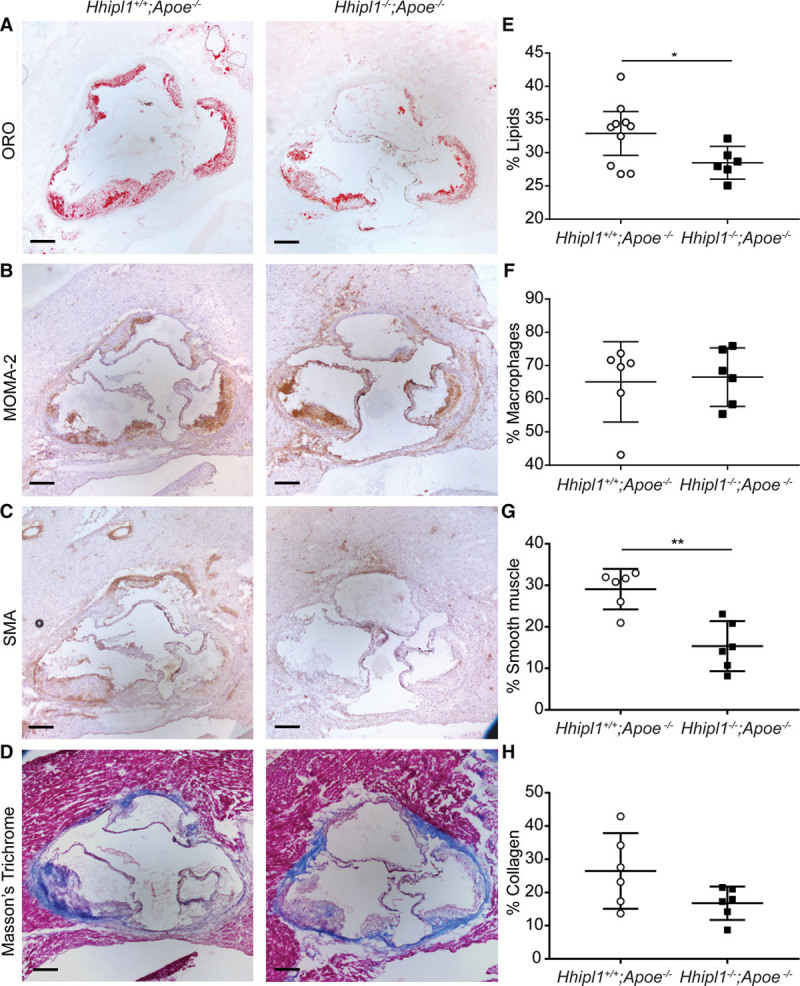
***Hhipl1* deficiency reduces smooth muscle cell content in *Hhipl1***^***−/−***^;***Apoe***^***−/−***^**atherosclerotic lesions.** Representative photomicrographs of atherosclerotic lesion components in *Hhipl1*^+/+^;*Apoe*^−/−^ and *Hhipl1*^−/−^;*Apoe*^−/−^ mice. **A**, Oil red O (ORO) staining for lipids; **C**, MOMA-2 staining for macrophages; **E**, anti-α smooth muscle actin (SMA) staining for smooth muscle cells; and **G**, Masson’s trichrome for collagen. Bars, 200 μm. Percentage coverage (average of 9 sections per animal) of (**B**) lipids, (**D**) macrophages, (**F**) smooth muscle cells (*P*=0.001), and (**H**) collagen (n=6–10 per group). Error bars represent mean±CI. HHIPL1 indicates hedgehog interacting protein-like 1. *P≤0.05, **P≤0.01. Bar, 200 μm.

We characterized the cellular and collagen composition of aortic root lesions from *Hhipl1*^−/−^;*Ldlr*^−/−^ (Figure 6A through 6H) and *Hhipl1*^−/−^;*Apoe*^−/−^ mice (Figure 7A through 7H) compared with *Hhipl1* wild-type littermates. Image analysis for lesion component coverage (as a percentage of the total lesion area) revealed no difference in lipids or macrophages within plaques of *Hhipl1*^−/−^;*Ldlr*^−/−^ mice compared with controls (Figure 6A through 6D). We detected a 46% reduction in cells stained for SMA in *Hhipl1*^−/−^;*Ldlr*^−/−^ lesions (13.9%; 95% CI, 9.7%–18.2%) compared with controls (25.6%; 95% CI, 20.9%, 30.5%; *P*=0.004; Figure 6E and 6F), as well as a nonsignificant reduction in collagen content in *Hhipl1*^−/−^;*Ldlr*^−/−^ (*P*=0.07; Figure 6G and 6H). We did detect a reduction in the lipid content of *Hhipl1*^−/−^;*Apoe*^−/−^ plaques (28.5%; 95% CI, 26%–31%) compared with controls (32.9%; 95% CI, 29.6%–36.2%; *P*=0.02; Figure 7A and 7B). The other components of *Apoe*^−/−^ plaques showed similar differences to those observed on the *Ldlr*^−/−^ background. There was no difference in macrophage staining (Figure 7C and 7D and a 47% reduction in SMA-positive staining in *Hhipl1*^−/−^;*Apoe*^−/−^ mice (15.3%; 95% CI, 9.3%–21.4%) compared with controls (29.1%; 95% CI, 24.2%–33.9%; *P*=0.001; Figure 7E and 7F) and a nonsignificant decrease in collagen content (*P*=0.08; Figure 7G and 7H). In addition to higher smooth muscle and collagen content, plaques from *Hhipl1*^−/−^ mice contained fewer cholesterol crystals and smaller lipid cores than controls (Figure IVA through IVF in the online-only Data Supplement).

## Discussion

A major challenge after GWAS is the identification of the causal gene and biological mechanisms underlying each disease-associated locus. In this study, we investigated *HHIPL1*, an uncharacterized gene at the chromosome 14q32 CAD locus, and showed that it encodes a secreted SHH regulator that modulates atherosclerosis-relevant smooth muscle cell phenotypes. We detected Hhipl1 expression in smooth muscle cells in atherosclerotic plaques, and its expression increased with disease progression. Most strikingly, knockout of *Hhipl1* caused a substantial decrease in atherosclerosis on 2 different disease-prone backgrounds. Our data strongly support *HHIPL1* as the causal gene at the 14q32 CAD locus, link hedgehog signaling to atherosclerosis, and identify HHIPL1 as a potential target for therapeutic intervention.

HHIPL1 is a paralog of the hedgehog signaling modulator HHIP,^5^ which interacts with each of the 3 hedgehog ligands and inhibits signaling.^6,11,12^ HHIP is associated with lung function and chronic obstructive pulmonary disease in humans,^33,34^ and homozygous knockout of *Hhip* in mice causes lethality because of abnormal lung development,^35^ whereas heterozygous knockout animals develop emphysema.^36^ The function of HHIPL1 has not been investigated previously. Our data demonstrate that HHIPL1, like HHIP, is a secreted hedgehog interacting protein; however, unlike HHIP, HHIPL1 positively regulates hedgehog signaling. In addition to its homology with HHIP, HHIPL1 also contains a C-terminal scavenger receptor cysteine-rich domain. This domain is present in some of the scavenger receptors involved in lipid uptake in plaque development and is thought to be involved in protein-protein interactions or ligand binding.^37^ Interestingly, cholesterol modification of the hedgehog proteins controls their distribution and receptor interactions.^38^ It is unclear whether the HHIPL1 scavenger receptor cysteine-rich domain is involved in the interaction with SHH via its cholesterol modification or some other mechanism, or whether it is required for a different hedgehog independent function.

We detected *HHIPL1* expression in AoSMCs and found that HHIPL1 protein localized to smooth muscle cells in atherosclerotic plaques. We also observed increased *Hhipl1* expression in the aortic roots of older *Apoe*^−/−^ mice. This is likely the result of increasing numbers of smooth muscle cells during atherosclerosis progression; however, we cannot exclude *Hhipl1* expression also being affected by other factors related to plaque development. In normal adult arteries, the core hedgehog proteins are expressed between the adventitial and medial layers^39^ and after injury in smooth muscle cells in the media and intima.^40,41^ Although our results suggest that smooth muscle is the primary site of HHIPL1 function, we cannot exclude a role for Hhipl1 in other atherosclerosis-relevant cell types, such as endothelial or inflammatory cells. Conditional knockout of Hhipl1 in smooth muscle will help determine the cell specificity of its role in disease pathogenesis.

*HHIPL1* deficiency reduced smooth muscle cell migration and proliferation in both human and mouse cells in vitro and reduced the proportion of smooth muscle cells in plaques in vivo. The hedgehog signaling pathway is an established regulator of cell behavior in multiple different systems,^7,42,43^ including smooth muscle cells.^22,40,41,44–47^ Hedgehog proteins can control these phenotypes through a variety of different mechanisms, including directly acting as chemoattractants and by inducing signaling pathways involved in cell-shape regulation and cell-cycle control. Although the exact mechanism of action of the hedgehog pathway on smooth muscle is unclear, SHH has been shown to mediate PDGFB (platelet-derived growth factor B)-induced smooth muscle migration via ERK and PI3K signaling,^22^ and hedgehog induction of neuropilins, which act as coreceptors of semaphorins and vascular endothelial growth factor, has been linked to cell migration in the development of the aortic arch.^48^ Previous studies have also demonstrated that inhibition of hedgehog signaling induces smooth muscle cell apoptosis.^45,47^ We did not detect a difference in apoptosis in either our mouse or human experiments, which is possibly a result of the moderate reduction in hedgehog signaling caused by *HHIPL1* deficiency compared with substantial loss of activity through pathway inhibition.

Our data clearly demonstrate a role for *HHIPL1* in atherosclerosis, with *Hhipl1* knockout reducing plaque burden by >50% in 2 different hyperlipidemic mouse models. This reduction was not attributable to any changes in body weight, blood, or plasma lipids. Previously, Beckers et al^29^ used a monoclonal antibody that inhibits all 3 hedgehog proteins to investigate atherosclerosis in *Apoe*^−/−^ mice and found that treated animals had larger, more advanced atherosclerotic plaques. The findings of that study are somewhat different from ours, because *Apoe*^−/−^ mice treated with the inhibitory antibody did not gain weight and had reduced plasma cholesterol levels, and the increase in atherosclerosis was driven by an increase in macrophage content, with no effect on smooth muscle. The efficiency of hedgehog inhibition in cells in the vessel wall was minimal, and the differences versus our study probably reflect the more general effects of global hedgehog inhibition. Nevertheless, both sets of data support a role for hedgehog signaling in atherosclerosis, and further investigation of the hedgehog pathway in disease pathogenesis and as a potential target for the treatment of CAD is warranted. Our data would suggest that inhibition of hedgehog signaling would reduce plaque development, and several hedgehog pathway inhibitors exist, including the SMO antagonists vismodegib and sonidegib, which have US Food and Drug Administration approval for treatment of basal cell carcinoma.^49,50^ Directly targeting HHIPL1 might also represent a promising option for therapy.

Current drug therapies for reducing atherosclerosis are primarily targeted toward lipids. Our findings suggest that targeting vascular smooth muscle cells may also be beneficial. Smooth muscle cell proliferation also plays a role in other vascular pathologies, including restenosis after percutaneous coronary intervention and coronary graft occlusion. Whether HHIPL1 plays a role in these conditions and whether targeting it would be of benefit remains to be determined.

In conclusion, HHIPL1, whose locus is associated with CAD in humans, is a new positive regulator of hedgehog signaling that promotes atherosclerosis in mice. Known hedgehog pathway modulators or novel therapeutic agents that directly target HHIPL1 are potential new treatments for CAD.

## Acknowledgments

We acknowledge the staff in the University of Leicester Division of Biomedical Services for technical expertise and animal care and the University of Leicester Core Biotechnology Services for assistance in plasmid construction (PROTEX) and image analysis (Advanced Imaging Facility). We thank Professor P.A. Beachy for providing the SHH-LIGHT2 cell line and Professor Martin Bennett for providing sections from *Apoe*^−/−^ mice. D.A., N.J.S., E.J.S., and T.R.W. designed the study. D.A., G.E.M., P.D.J., and H.K.T. performed experiments. E.K., and R.B.K. contributed to immunohistochemical analysis. M.A.K., M.N.G., and S.L.A contributed to cell-based experiments. D.A., and M.N. performed statistical analyses. Data analysis and interpretation was performed by D.A., G.E.M., P.D.J., H.K.T., M.N., S.Y., N.J.S., E.J.S., and T.R.W. All authors contributed to the writing and preparation of the manuscript.

## Sources of Funding

The research leading to these results has received funding from the European Union Seventh Framework Programme FP7/2007–2013 under grant agreement number HEALTH-F2-2013–601456, a Transatlantic Networks of Excellence Award (12CVD02) from The Leducq Foundation and the British Heart Foundation (SP/18/8/33620) as a partner of the European Research Area Network on Cardiovascular Diseases (ERA-CVD) druggable-MI-genes (01KL1802) and supported by the UK National Institute for Health Research (NIHR) Leicester Biomedical Research Centre. Dr Samani is a UK NIHR Senior Investigator. Drs Morris, Ye, and Webb are funded by the British Heart Foundation (SP/16/4/32697).

## Disclosures

None.

## Supplementary Material
