# Supplementary material for: HHIPL1, a Gene at the 14q32 Coronary Artery Disease Locus, Positively Regulates Hedgehog Signaling and Promotes Atherosclerosis
Source: Circulation. 2019 Jun 5;140(6):500–13. doi: 10.1161/CIRCULATIONAHA.119.041059 (PMC6686954; doi:10.1161/CIRCULATIONAHA.119.041059)
Supplement: Supplementary file 1 [file cir-140-500-s001.pdf]

SUPPLEMENTAL MATERIAL

Supplementary Extended Methods

Plasmid constructs: Full-length human HHIPL1 cDNA (Genscript, NM_001127258) was cloned into the C-terminal GFP-tag plasmid pLEICS-29 and the C-terminal FLAG-tag plasmid pLEICS-49. Full-length human SHH (pANT7_cGST plasmid, HsCD00005768, DNASU) was cloned into pLEICS-29 by the Protein Expression Laboratory (PROTEX) cloning service at the University of Leicester.

Transfection, immunoprecipitation and western blotting: HEK293 (ATCC CRL-1573) cells were transiently transfected with plasmid DNA using either a NEPA21 electroporator (NEPA gene) or Lipofectamine 2000 (ThermoFisher). Immunoprecipitation was performed using GFP-TRAP beads (Chromotek). Samples were resolved alongside pre-immunoprecipition samples and western blotting performed: Cell lysates were collected in HEPES lysis buffer supplemented with protease and phosphatase inhibitors and denatured in 1X NuPAGE LDS containing 20 µM DTT. Conditioned media proteins were precipitated by addition of an equal volume of 20% trichloroacetic acid. Protein pellets were washed in pure acetone and resuspended in 1X NuPAGE LDS containing 20µ M DTT. Proteins were resolved on pre-cast polyacrylamide NuPAGE Novex 10% Bis-Tris gels (ThermoFisher) and transferred to a nitrocellulose membrane by wet-transfer using the XCell II Blot module (Novex, ThermoFisher). Antibodies used for immunoblotting: Anti-FLAG (Sigma Aldrich F3165), Anti-GFP (ThermoFisher Scientific MA5-15256), (Anti-GLI1 (R&D Systems AF3455), Anti--β-actin (ThermoFisher Scientific, 11355703), Anti-mouse IgG-HRP (Amersham NA931), Anti-rabbit IgG- HRP (Amersham NA934). Blots were visualized by chemilluminescence on an ImageQuant LAS 4000 (GE healthcare).

Immunofluorescence: HEK293 cells transfected with HHIPL1-GFP plasmid were seeded on coverslips. 48 hours post transfection the cells were fixed in 4% (*w/v*) paraformaldehyde (PFA) for 10 minutes followed by permeabilisation with 0.2% Triton- X. Nuclei were counterstained stained with DAPI (ThermoFisher Scientific). Cells were visualized by an Olympus FV1000 confocal microscope (University of Leicester Advanced Imaging Facility).

Preparation of mouse AoSMCs: Mouse AoSMCs were prepared from aortas of *Hhipl1*$^{-/-}$ mice and wild-type littermates. Aortas were removed and cleaned of connective tissue, and transferred to basal Dulbecco's Modified Eagle's Medium (DMEM) containing collagenase type 2 (1 mg ml-1) and warmed to 35 °C for 10 minutes. Aortic adventitia was removed prior to the aorta being transfer to DMEM containing collagenase type 2 (1.36 mg ml^{-1}) and elastase (7 mM (0.18 mg ml^{-1})) for 90 minutes. Single smooth muscle cells were obtained by trituration with a wide-bore pipette and were washed by centrifugation. Mouse AoSMCs were maintained in DMEM supplemented with 20% foetal calf serum (FCS), 1% antibiotic solution (10,000 units/ml penicillin G, 100 mg ml^{-1} streptomycin sulfate) at 37 °C and 5% CO_2 in humidified conditions. After two passages cells were cultured in DMEM containing 10% FCS. Cells between passage 3 and 7 were used in all experimentation.

Preparation of human macrophages: Human monocytes and macrophages were purified from the blood of healthy individuals using the protocol described previously [1]. Briefly, peripheral blood mononuclear cells (PBMCs) were centrifuged over lymphoprep™ (STEMCELL Technologies) at 800 g for 30 minutes. The mononuclear cells were extracted from the interphase and washed several times before being seeded and incubated on 6 well plates in a humidified incubator at 37°C and 5% CO2 in serum free RPMI-1640 media for 2 hours. Cells were washed exstensively, and adherent monocytes were cultured in macrophage-serum

free media with additional human macrophage colony stimulating factor (50 ng/ml) for a further 5 days before cellular RNA was extracted.

Expression analysis: Mouse *Hhipl1* gene expression (forward primer: 5'-ACTTCATGTCCACGGGTGTT-3' and reverse primer: 5'-CTCCTCACCTTCACCTGAGC-3') was measured in cDNA prepared from the aortic arch and the thoracic aorta of wild-type C57BL/6J and *Apoe*$^{-/-}$ mice between 6 and 48 weeks of age by quantitative real time PCR (qRT-PCR) using the Rotor-Gene system (Qiagen). *Ptch1* (forward primer: 5'-ATGCTCCTTTCCTCCTGAAACC-3' and reverse primer: 5'- TGAACTGGGCAGCTATGAAGTC-3') and *GLI1* (forward primer: 5'- CCAAGCCAACTTTATGTCAGGG-3' and reverse primer: 5'-AGCCCGCTTCTTTGTTAATTTGA-3') expression was measured in cDNA prepared from three wild-type and three *Hhipl1* knockout aortic smooth muscle cells (AoSMCs). Comparative quantitation was performed using the Rotor-Gene software. For each reaction an appropriate sample was set as calibrator and in order to correct for variation in starting amount and all samples were normalised to either *Rpl4* (forward primer: 5'-CGCAACATCCCTGGTATTACT-3' and reverse primer: 5'-ACTTCCGGAAAGCACTCTCCG-3') or *Rplp0 (36B4)* (forward primer: 5'-TGAAGCAAAGGAAGAGTCGGAGGA-3' and reverse primer: 5'-AAGCAGGCTGACTTGGTTGCTTTG-3'). For expression analysis in human cells the primers used are as follows: *HHIPL1* (forward primer: 5'-ACTTCATGTCCACGGGTGTT, reverse primer: 3'-CTCCTCACCTTCACCTGAGC), *GLI1* (forward primer: 5'-CCAAGCCAACTTTATGTCAGGG, reverse primer: 3'-AGCCCGCTTCTTTGTTAATTTGA), *PTCH1* (forward primer: 5'-ATGCTCCTTTCCTCCTGAAACC, reverse primer: 3'-TGAACTGGGCAGCTATGAAGTC). Expression was normalised to *RPLP0* (forward primer: 5'-TGAAGCAAAGGAAGAGTCGGAGGA, reverse primer: 3'-AAGCAGGCTGACTTGGTTGCTTTG).

Cellular assays: Human AoSMCs (ThermoFisher) were reverse transfected with 50 nM siRNA (Qiagen, Hs_KIAA1822_1, Hs_HHIPL1_2) against *HHIPL1* or a non-targeting control siRNA using RNAiMAX transfection reagent (ThermoFisher). Mouse AoSMC proliferation was determined by incubating with PrestoBlue® (ThermoFisher) and measuring fluorescence emission on a plate reader (excitation 560 nm/emission 590 nm) and normalized to 0 hour time point to account for variation in fluorescence intensity between independent experiments. Human AoSMC proliferation was performed by counting cells. Each time point comprises the average cell number across 3 images in each of 3 technical replicates across 4 independent experiments. Cell migration was measured using a wound-healing assay. Cells were grown in a 24-well plate until confluent, then a scratch created across the whole well using a pipette tip. Three images were collected per well every two hours for 12 hours using an EVOS Microscope (Advanced Microscopy Group). Cell coverage was assessed using ImageJ. The proportion of apoptotic cells was determined by staining with FITC-Annexin-V and measured using a Beckman Coulter Gallios flow cytometer.

Blood pressure measurement: Mouse restraint tubes were put into the home cages at least 24 hours before blood pressure measurement in order for the mice to acclimatise. Conscious mice were placed into the holders and the systolic and diastolic blood pressure was measured using a tail-cuff system (CODA-4.0 equipment, Kent Scientific Corporation, Connecticut, USA) according to the manufacturer's instructions. A standard program of 5 acclimatisation cycles followed by 15 cycles of measurements (with 5 sec between cycles and a deflation time of 15 sec) was used.

Lipid measurement: Blood was collected by saphenous vein puncture at 6 weeks of age and from the vena cava at the end of the experiment 12 weeks later. Blood was left for 30 minutes at room temperature to clot and plasma obtained by centrifugation and stored at -80 °C. The

levels of total cholesterol, low-density lipoprotein (LDL)/very low-density lipoprotein (VLDL) cholesterol and high-density lipoprotein (HDL) cholesterol in plasma was using an enzymatic colorimetric assay (ab65390; Abcam) according to the manufacturer's protocol. Briefly, separation of HDL and LDL/VLDL cholesterol was performed by mixing 10 µl plasma with 10 µl 2X precipitation buffer. Following centrifugation, supernatant (HDL fraction) was transferred to a new tube and the precipitate was resuspended in PBS (LDL/VLDL fraction). Absorbance of each fraction was read at 570nm and final data were extrapolated from a standard curve.

Supplementary References

1. Morris GE, Braund PS, Moore JS, Samani NJ, Codd V, Webb TR. Coronary Artery Disease-Associated LIPA Coding Variant rs1051338 Reduces Lysosomal Acid Lipase Levels and Activity in Lysosomes. Arterioscler Thromb Vasc Biol. 2017;37(6):1050-1057. doi: 10.1161/ATVBAHA.116.308734.

Supplementary Figures

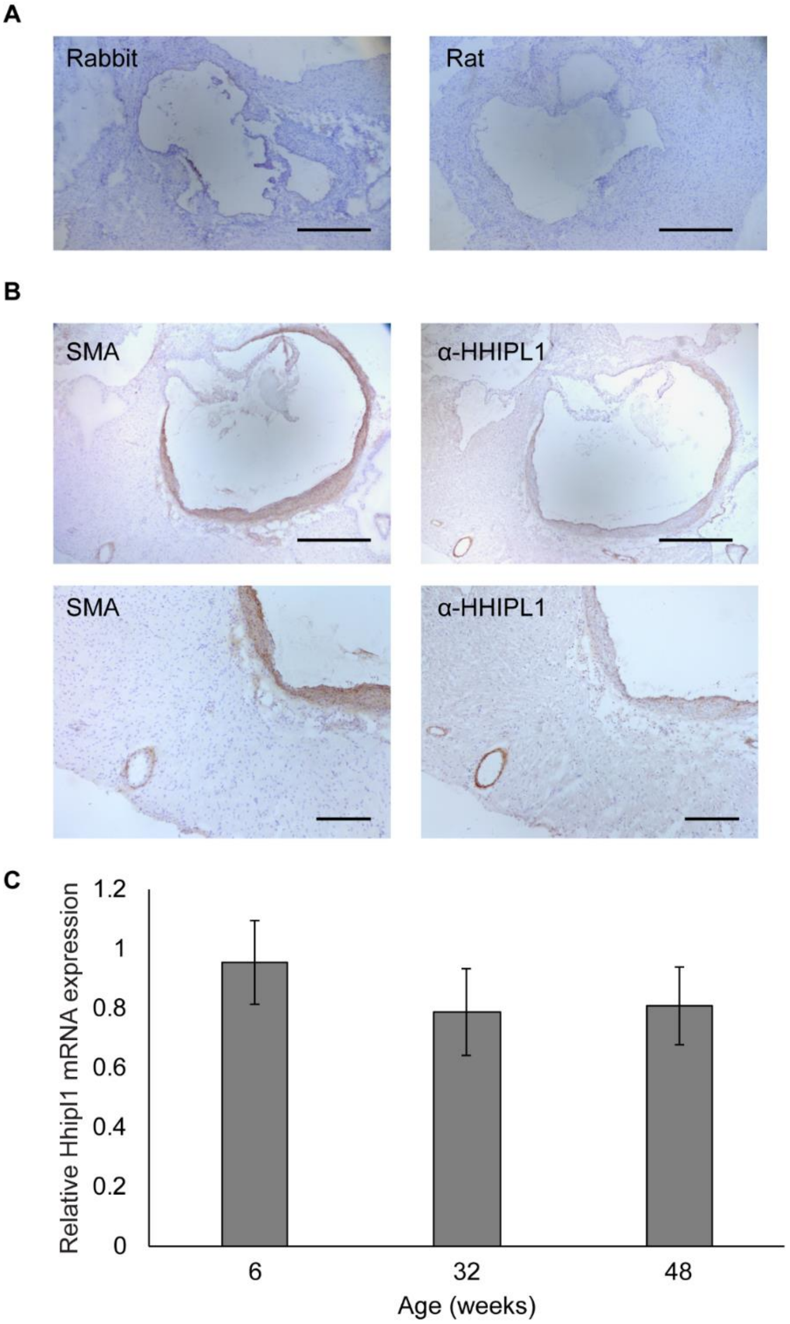

Figure S1: Hhipl1 expression in aortic arches of wild-type mice

(**A**) No primary antibody controls. scale bars = 500 µm (**B**) Representative immunohistochemical staining with anti-alpha smooth muscle Actin antibody (SMA) or anti-

Hhipl1 in wild-type mice. Upper panel scale bars = 500 µm. Lower panel scale bars = 200 µm. (**C**) *Hhipl1* mRNA expression relative to *Rpl4* in the aortic arch of of 6-48 week old wild-type mice. n=3-9 mice per time-point.

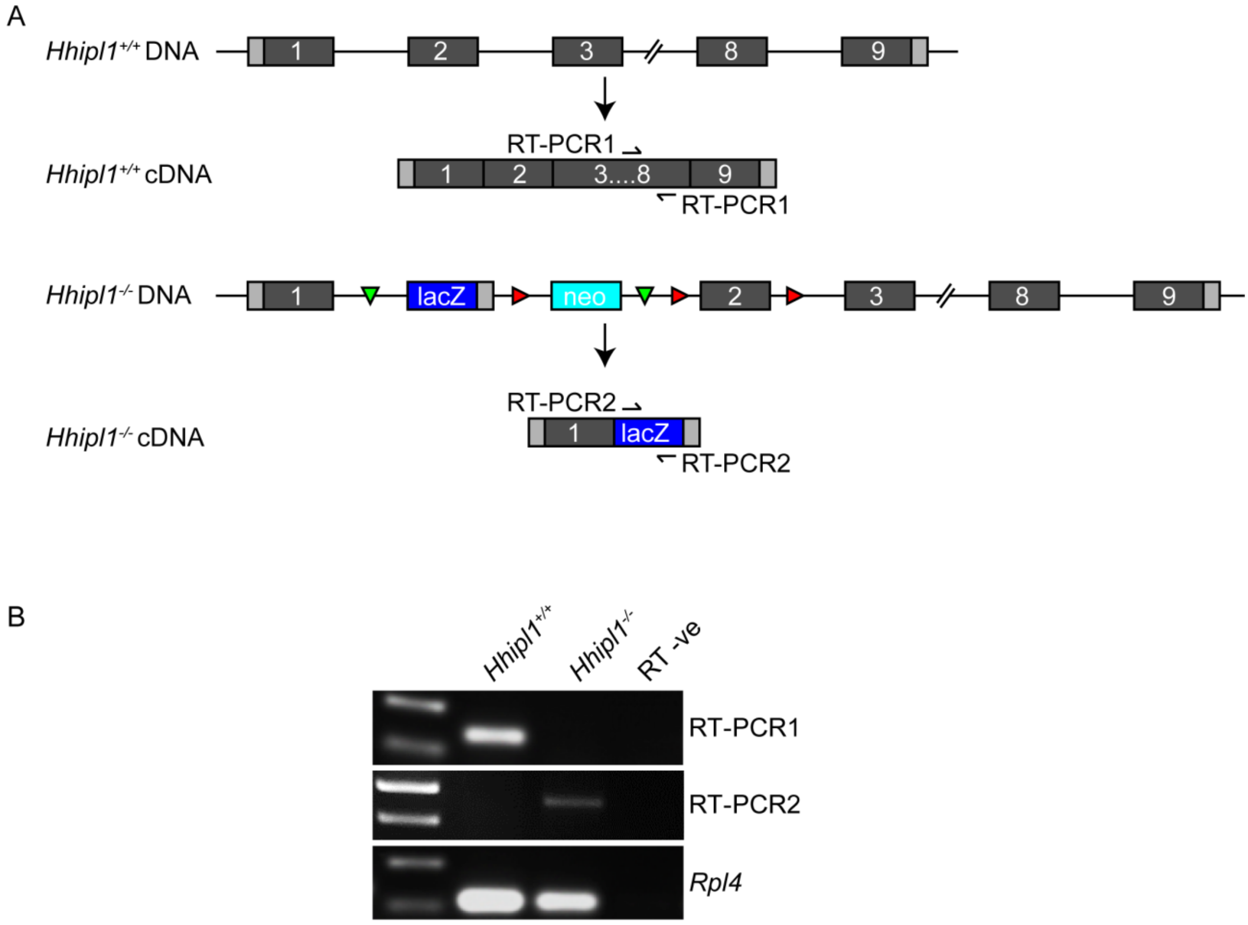

Figure S2: Generation of knockout mice

(**A**) Schematic representation of the wild type and *Hhipl1*$^{-/-}$ alleles at the DNA and cDNA level. Green triangles: FRT sites, Red triangles: *lox*P sites. Location of primer pairs for RT-PCR1 and RT-PCR2 are shown as labelled arrows. (**B**) RT-PCR analysis using RT-PCR1 and RT-PCR2 primer to amplify cDNA prepared from *Hhipl1*$^{+/+}$ and *Hhipl1*$^{-/-}$ mice. *Rpl4* served as a loading control.

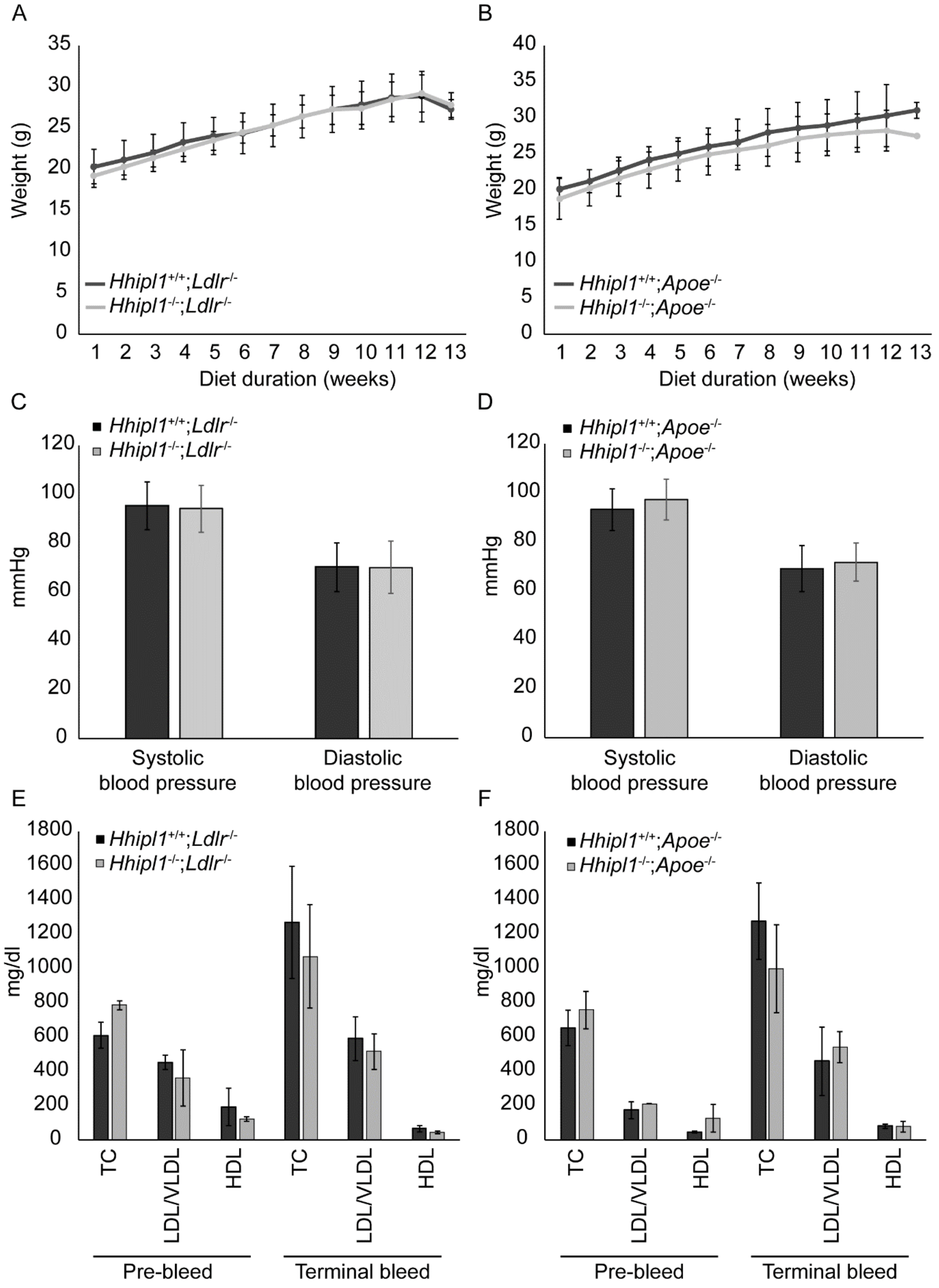
A
Weight (g)
Hhipl1+/+;Ldlr-/-
Hhipl1-/-;Ldlr-/-
Diet duration (weeks)
B
Weight (g)
Hhipl1+/+;Apoe-/-
Hhipl1-/-;Apoe-/-
Diet duration (weeks)
C
Hhipl1+/+;Ldlr-/-
Hhipl1-/-;Ldlr-/-
mmHg
Systolic blood pressure
Diastolic blood pressure
D
Hhipl1+/+;Apoe-/-
Hhipl1-/-;Apoe-/-
mmHg
Systolic blood pressure
Diastolic blood pressure
E
Hhipl1+/+;Ldlr-/-
Hhipl1-/-;Ldlr-/-
mg/dl
TC
LDL/VLDL
HDL
Pre-bleed
Terminal bleed
F
Hhipl1+/+;Apoe-/-
Hhipl1-/-;Apoe-/-
mg/dl
TC
LDL/VLDL
HDL
Pre-bleed
Terminal bleed

Figure S3: Analysis of body weight, blood pressure and plasma lipid levels

(**A** and **B**) Body weight was measured weekly during the atherosclerosis study (n=17-19 per group). (**C** and **D**) Systolic and diastolic blood pressure measurements (n=15-19 per group). (**E** and **F**) Results of total cholesterol, VLDL/LDL and HDL measurements performed at the start and end of the atherosclerosis study. No statistically significant difference was identified between *Hhipl1*$^{+/+}$ and *Hhipl1*$^{-/-}$ mice on either hyperlipidemic background in any experiment.

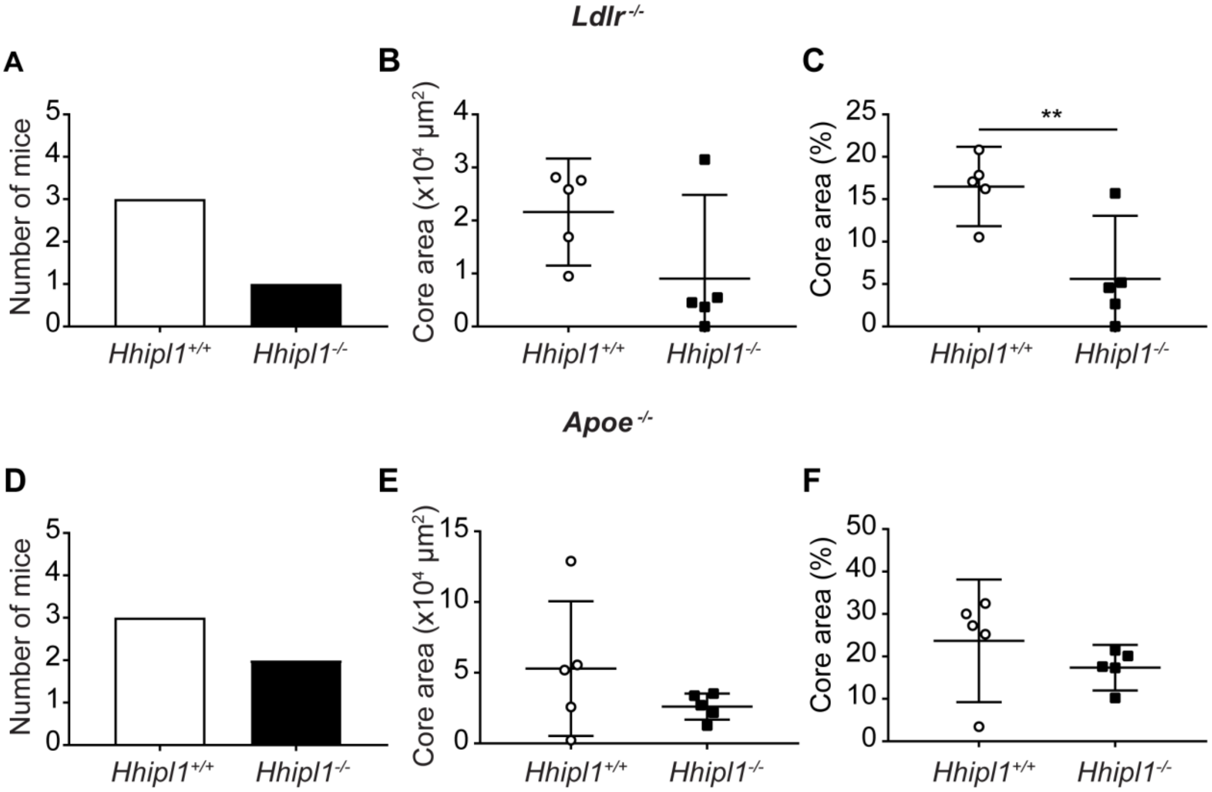

Figure S4: Plaque characteristics of *Hhipl1* knockout mice.

(**A** and **D**) Number of mice with plaques containing cholesterol crystals. (**B** and **E**) Lipid core area. (**C** and **F**) Lipid core as a proportion of plaque area. N=5 per group. Error bars represent mean ± 95% CI. ***P*≤ 0.01.
